# Supplementary material for: Caring for the Elderly Enhances Positive Attitudes Better Than Knowledge in Nursing Students
Source: Medicina (Kaunas). 2022 Sep 1;58(9):1201. doi: 10.3390/medicina58091201 (PMC9506512; doi:10.3390/medicina58091201)
Supplement: Supplementary file 1 [file medicina-58-01201-s001.zip › medicina-1874376-supplementary.pdf]

Table S1. Items included in Kogan's scale.

Item	Type	Statement of the question
1	Negative	It would be better if most of the elderly lived with their coevals in the same place
2	Positive	It would be better if most of the elderly lived in places where the young also lived
3	Negative	There is something different with most of the elderly: it is difficult to understand what makes them restless/tick
4	Positive	Most of the elderly are not different from anybody; understanding them is as easy as understanding the young
5	Negative	Most of the elderly live as they wish and cannot change
6	Positive	Most of the elderly can adapt themselves to the changes required by the conditions
7	Negative	Most of the elderly prefer to get retired as soon as entitled to it or their children are able to look after them
8	Positive	Most of the elderly would like to continue to work as long as possible rather than be dependent on anybody
9	Negative	Most of the elderly tend to let their houses untidy and unkempt
10	Positive	Most of the elderly can keep their houses clean and tidy
11	Negative	It is foolish to say that wisdom comes by old age
12	Positive	People grow wiser with coming of old age
13	Negative	The elderly have too much power in business life and politics
14	Positive	The elderly should have power in business life and politics
15	Negative	Most of the elderly easily make people ill/make them feel unwell
16	Positive	It is quite relaxing to be with the elderly
17	Negative	Most of the elderly bore others by talking about "good old days"
18	Positive	One of the most interesting and entertaining qualities of most elderly people is to tell about their past experiences
19	Negative	Most of the elderly spend too much time mixing into other people's business (stick their noses in) and giving unsought advice
20	Positive	Most of the elderly tend to keep their opinions to themselves and give advice only when asked
21	Negative	If elderly people expect to be liked, their first step is to try to get rid of their irritating faults
22	Positive	When you think about it, old people have the same faults as anybody else
23	Negative	It's best to move to an area where there not many elderly people in order to keep your neighborhood nice
24	Positive	You can be sure to find a nice atmosphere if there is a sufficient number of elderly people in your neighborhood
25	Negative	There are a few exceptions, but in general most old people are pretty much alike
26	Positive	It is evident that most old people are very different from one another
27	Negative	Most of the elderly should take care of their personal appearance; they are too untidy
28	Positive	Most of the elderly have a clean and tidy personal appearance
29	Negative	Most of the elderly are irritable, grouchy and unpleasant
30	Positive	Most of the elderly are cheerful, agreeable and good humored
31	Negative	Most of the elderly constantly complaining about the behavior of the younger generation
32	Positive	Most of the elderly rarely complaining about the behavior of the younger generation
33	Negative	Most of the elderly need more love and reassurance than other people
34	Positive	Most of the elderly need as much love and reassurance as other people
